# The contribution of culturomics to the repertoire of isolated human bacterial and archaeal species

**DOI:** 10.1186/s40168-018-0485-5

**Published:** 2018-05-24

**Authors:** Melhem Bilen, Jean-Charles Dufour, Jean-Christophe Lagier, Fréderic Cadoret, Ziad Daoud, Grégory Dubourg, Didier Raoult

**Affiliations:** 1 IRD, APHM, MEPHI, IHU Méditerranée Infection, Aix-Marseille Université, Marseille, France; 2 Clinical Microbiology Laboratory, Faculty of Medicine and Medical Sciences, University of Balamand, PO Box: 33, Amioun, Lebanon; 3 INSERM, IRD, SESSTIM UMR912, Aix-Marseille Université, Marseille, France; 4 Assistance Publique Hôpitaux de Marseille, BIOSTIC Service Biostatistique et Technologies de l’Information et de la Communication, Hôpital de la Timone, Marseille, France; 5 Special Infectious Agents Unit, King Fahd Medical Research Center, King Abdulaziz University, Jeddah, Saudi Arabia; 6 Microbes, Evolution, Phylogeny and Infections (MEPHI), AMU, IRD, Institut Hospitalo-Universitaire Méditerranée-Infection, Aix-Marseille Université, 19-21 Boulevard Jean Moulin, 13385 Marseille CEDEX 5, France

**Keywords:** Human microbiota, Culturomics, Gut, Isolation, Prokaryotes, New species

## Abstract

After a decade of research and metagenomic analyses, our knowledge of the human microbiota appears to have reached a plateau despite promising results. In many studies, culture has proven to be essential in describing new prokaryotic species and filling metagenomic gaps. In 2015, only 2172 different prokaryotic species were reported to have been isolated at least once from the human body as pathogens or commensals. In this review, we update the previous repertoire by reporting the different species isolated from the human body to date, increasing it by 28% to reach a total of 2776 species associated with human beings. They have been classified into 11 different phyla, mostly the *Firmicutes*, *Proteobacteria*, *Bacteroidetes*, and *Actinobacteria*. Finally, culturomics contributed up to 66.2% towards updating this repertoire by reporting 400 species, of which 288 were novel. This demonstrates the need to continue the culturing work, which seems essential in order to decipher the hidden human microbial content.

**Electronic supplementary material:**

The online version of this article (10.1186/s40168-018-0485-5) contains supplementary material, which is available to authorized users.

## Background

This review reports the different bacterial species isolated at least once from the human being and emphasizes on the contribution of culturomics in unveiling and describing the human microbiota.

## Introduction

Prokaryotes are known for their abundance, diversity, and presence in almost all types of niches, as well as their ability to live in different environmental conditions [1–3]. Despite more than a century of research in microbiology, estimating prokaryotic diversity remains a challenge. For instance, scientists estimate a range of between 10^7^ and 10^12^ species per 1 g of stool, but few have been isolated by culture [4, 5]. However, the human microbiota composition has been strongly correlated with health and diseases where the balance of residing bacterial population was shown to be associated with an array of pathologies [6–13]. This highlighted the fact that the human microbiota can be used in therapeutic approaches, such as probiotic design or commensal replenishment [14–17] and the need to decipher its content for a better global understanding. Nevertheless, several approaches are currently being used in order to identify and describe the human microbiota. Culture is the oldest technique used for growing and isolating bacterial colonies. With the advances in sequencing techniques, particularly metagenomics, which targets the genetic material that can be recovered from any sample, the scientific community started believing that culture would no longer be needed [18, 19]. Yet, it soon appeared that these new methods have multiple drawbacks, such as depth bias and inability to detect, in some cases, the causative pathogenic bacteria such as the investigation into a shigatoxigenic *Escherichia coli* (STEC) O104:H4 outbreak [20] or *Campylobacter* and *Salmonella* in diarrhea cases [21]. In addition, incomplete genomic databases make it difficult to assign a precise taxonomic rank to a substantial number of sequences. Most importantly, these methods do not provide a pure culture of microorganisms, which is crucial for further strain characterization, in vitro models, or host-interaction studies [22]. Culturomics was introduced in order to optimize culture conditions and to show that the term “uncultivable organism” is misleading, since all microorganisms are cultivable using the right conditions and tools [18]. Besides the different culture conditions used, culturomics is coupled with an efficient, fast, and cost-effective identification methodology, based on MALDI-TOF MS and 16S rRNA gene sequencing, in the event of MALDI-TOF MS identification failure [18]. The complementarity between culture-independent and culture-dependent studies was well established, since only 15% of the detected species were concurrent for these two techniques [19, 23–25]. Culturomics has played an important role in the description of the human microbiota, particularly to fill the gaps of metagenomics by attributing several OTUs (operational taxonomic units) from other studies to newly isolated bacterial species [26].

A repertoire of all prokaryotes isolated by culture, at least once from the human body, was created in 2015 [27]. This review aims to update the repertoire of prokaryotes that have been isolated from different sites on the human body and complements the work published by Hugon et al. in 2015, who reported 2172 different species [27]. Additionally, it aims to highlight the contribution of culturomics in describing the human microbiota.

## Culturomics: a strategy complementing metagenomics for describing the human gut microbiota

Metagenomic studies have reintroduced the study of the human gut microbiota through large amounts of generated data. Few of these studies have proved however to be significant [28–30] due to multiple factors, such as the inappropriate data analyses (i.e., bioinformatics, statistical analysis) or study design (i.e., inappropriate controls, small sampling size). Several studies have shown the ability to isolate bacterial species by culture, those very species that were not detected by high-throughput sequencing methods. For example, in 2013, Dubourg et al. showed that the number of bacterial species isolated by culture from stool samples may outrange the results of pyrosequencing [23]. Moreover, in some studies, the proportion of gram-positive to gram-negative bacteria, when comparing culture to sequencing results, revealed some differences [31–33]. Hayashi et al., in 2002, studied the digestive microbiota by the means of cloning/sequencing and anaerobic culture and revealed discrepancies between these approaches since the results were not equivalent [34]. Similar research comparing these two techniques when studying the human colonic microbiota also revealed that the reported species by culture or metagenomics were discordant with a significant number of bacterial species isolated by culture not identified by metagenomics [35]. In addition, Lagier et al., in 2012, have studied the bacterial content of three stool samples and highlighted an inconsistency between culturomics and pyrosequencing of 16S rRNA gene amplicons, where only 15% of the reported species were mutual between the two techniques [36]. These facts brought to interest the culture of microbiota with Lagier et al. in 2015. Using culturomics, 1057 prokaryotic species were identified, subsequently adding 531 species to the human gut repertoire, including 146 species which were previously isolated in humans but not in the gut, one archaea, 187 species which were not previously identified in humans, and 247 new species [26]. Additionally, taxonomic assignment, at the species level, was made possible for a significant number of unassigned sequences generated by previous metagenomic studies when comparing it to the genome sequences of the new species isolated by culturomics [26]. Thus, these two techniques complement one another and should be used together in order to more efficiently describe the human microbiota and reveal its dark matter.

## Expanding the human bacterial repertoire

### Methods

In 2015, our laboratory has previously established a repertoire of listing all bacteria isolated at least once from the human body between 1980 and February 16, 2015 (http://hpr.mediterranee-infection.com/arkotheque/client/ihu_bacteries/recherche/index.php) [27]. Thus, we have used the same methodology which included the three most sensitive queries selected, tested previously by a gold standard [27], to build a customized software using NCBI E-utilities to programmatically query the MeSH database, PubMed/Medline, and NCBI Taxonomy. With the means of this software, advanced searches on PubMed/Medline were made possible using a combination of NCBI taxonomy bacterial synonyms, MeSH keywords, abstract or title, and phylum information. Therefore, when using a specific bacterial species, the query pattern will allow us to retrieve the articles with their PMID that corresponds to its isolation from human [27]. In this review, we update the content of this repertoire, taking into consideration all the bacteria isolated by culture at least once from the human body and which have been reported by the List of Prokaryotic names with Standing in Nomenclature (LPSN) or NCBI taxonomy. Articles were collected from 2014 to April 17, 2018 and manually verified by title, abstract, and full text if needed, in order to confirm the results of the queries and that the article reports isolation of a bacterial species from humans. As for the pathogenicity pattern evaluation of the reported species in this repertoire, we investigated each species in the Risk Group Database (https://my.absa.org/Riskgroups) and reported any missing species as “unclassified.” Finally, in order to evaluate the oxygen requirement of the reported species, we used the database in http://www.mediterranee-infection.com/article.php?laref=374, and the taxonomic lineage was performed with the help of NCBI taxonomy (https://www.ncbi.nlm.nih.gov/taxonomy).

### The human bacterial repertoire: where do we stand?

**Fig. 1:**
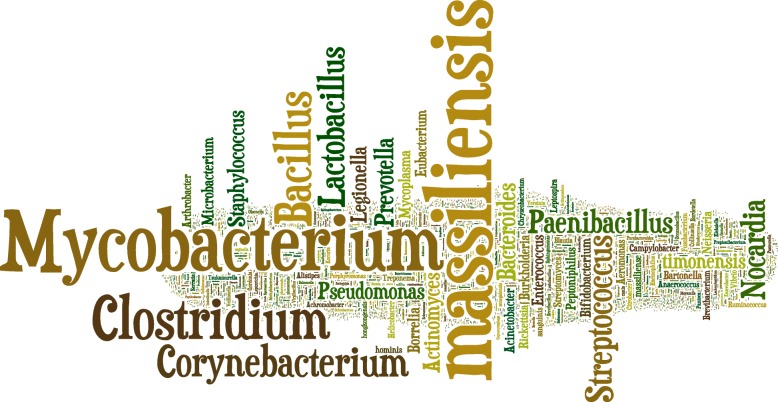
All 2776 species isolated at least once from humans by culture. Using the online tool wordle (www.wordle.net), the size of the name of each species is proportional to the number of times it occurs in the repertoire

**Fig. 2:**
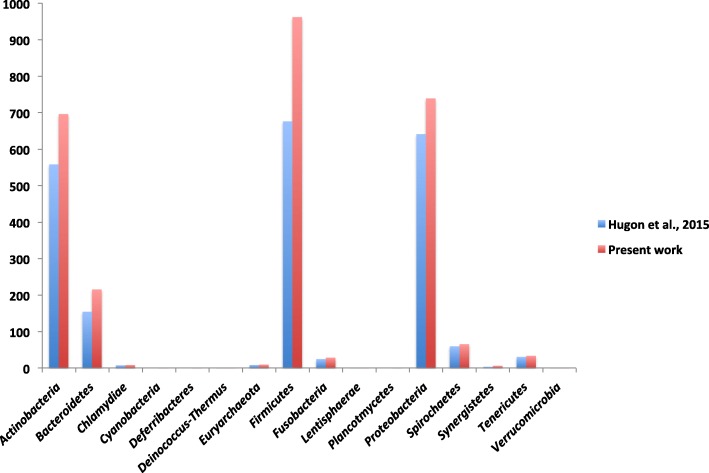
Distribution of bacterial species reported in this repertoire according to their phylum, based on the NCBI taxonomy classification with the highest category being clustered in the *Firmicutes* phylum. *Cyanobacteria*, *Deferribacteres*, *Spirochaetes*, *Synergistetes*, and *Tenericutes* represent minority phyla in this repertoire, with 1, 1, 1, 3, 3, and 3 different species, respectively

We added 604 different species to the previously reported human microbiota repertoire [27] (Additional files 1 and 2). This represents an increase of 28% in the number of species isolated from the human body [27] (Additional file 3). By adding our data to those reported in 2015 by Hugon et al., we reach a total of 2776 species isolated at least once from the human body (Fig. 1 and Additional file 3). Most of the added species belonged to the *Firmicutes*, *Actinobacteria*, and *Proteobacteria* phylum with 289 (47.76%), 137 (22.64%), and 99 (16.36%) species, respectively [27] (Table 1). The number of species isolated in *Chlamydiae*, *Deinococcus-Thermus*, *Lentisphaerae*, and *Verrucomicrobia* remained the same (Table 1). Interestingly, *Leptolyngbya ramose* and *Deferribacter desulfuricans* became the first members of the *Cyanobacteria* and *Deferribacteres* phylum, respectively, to be isolated from human beings in clinical cases [27] (Fig. 2, Additional files 1, 2, and 3, Table 1).

**Table 1:** Summary of all the results obtained in combination between Hugon et al.’s previous work and the present repertoire. Pathogenicity is represented as previously mentioned according to its risk group. Risk group 1 category refers to no or low individual and community risk, risk group 2 refers to species with moderate individual risk and a low community risk, risk group 3 represents species with high individual risk and a low community risk, risk group 4 being of a high individual and community risk, and unclassified refers to species that were not found in the risk group database

	Hugon et al., 2015 [27]	Present work
Risk group 1	2042	2042
Risk group 2	103	112
Risk group 3	27	28
Risk group 4	0	0
Unclassified	0	594
Strictly anaerobic total	386	662
Strictly anaerobic gut	244	459
*Actinobacteria*	558	696
*Bacteroidetes*	155	216
*Chlamydiae*	8	9
*Cyanobacteria*	0	1
*Deferribacteres*	0	1
*Deinococcus-Thermus*	1	1
*Euryarchaeota*	9	10
*Firmicutes*	676	962
*Fusobacteria*	25	29
*Lentisphaerae*	1	1
*Proteobacteria*	641	740
*Spirochaetes*	60	66
*Synergistetes*	4	7
*Tenericutes*	31	34
*Verrucomicrobia*	1	1
Unclassified	2	2
Total	2172	2776

**Fig. 3:**
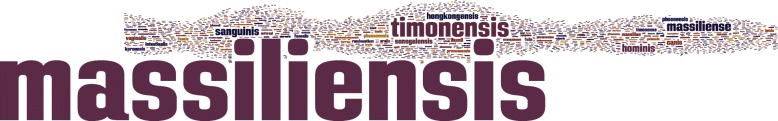
All 2776 species isolated at least once from humans using culture. Using the online tool wordle (www.wordle.net), the size of the name of each species is proportional to the number of times it occurs in the database. In this image, only the species name was taken into consideration, excluding the genus

Nevertheless, when looking at the genus level, it is clear that *Mycobacterium* is the most common of all 602 unique genera of the 2776 species, with 151 different species belonging to it. *Mycobacterium* belongs to the *Actinobacteria* genus and is also known to cause several serious diseases in humans, including tuberculosis and leprosy [37, 38]. Recently, a novel *Mycobacterium* species (*Mycobacterium saopaulense*) was isolated from a clinical specimen from a patient who underwent LASIK surgery [39]. This shows the importance of further work on culture in order to complement our understanding of the different bacterial species that may be pathogenic but have not yet been isolated or reported. Our laboratory contributed to the identification of 13 new species of *Mycobacterium* [40–48] that have been isolated from the respiratory tract, blood, gut, femur bone, and skin. As for the most frequently occurring species epithet in this repertoire, these were *massiliensis* (162) and *timonensis* (40) (Fig. 3), both of which have been reported by our laboratory and assigned to the novel bacterial species isolated by culturomics.

### The gut microbiota: richness and diversification

**Fig. 4:**
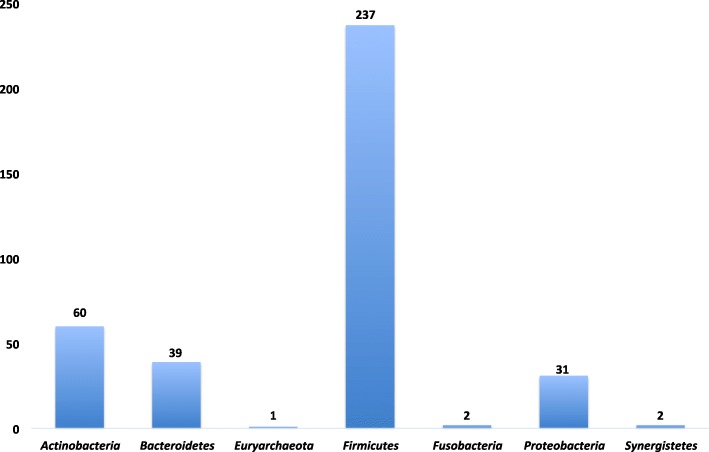
Distribution of bacterial species isolated from the gut using culturomics, according to the phylum to which they belong, based on the NCBI taxonomy classification, with the highest clustering in the *Firmicutes* phylum

The largest human microbiota resides in the gut, and only the human colon is known to have a bacterial density of more than 10^10^ cells/g [49]. Previous studies have measured around 10 million different genes in the human gut microbiome [50] and more than 1000 species belonging to several phyla, with the most common being *Bacteroidetes*, *Firmicutes*, and *Actinobacteria*, while *Fusobacteria*, *Cyanobacteria*, *Proteobacteria*, and *Verrucomicrobia* are less frequently encountered [51, 52]. Of the 604 species reported in this study, 372 species were isolated in the gut, of which 92.5% were isolated by culturomics, including 232 new species (Additional files 1 and 2 and Fig. 4).

### The significant contribution of anaerobes

Because they are unable to tolerate or grow in the presence of oxygen, the distribution of anaerobic bacteria in the human body will vary by sites. However, it is expected and has been demonstrated that these organisms are more highly concentrated in the digestive tract compared to the skin, vagina, or oral cavities [53, 54]. Culturing and isolating these species is challenging and requires special conditions. There is also a slow replication rate. In recent years, various anaerobic methods have been improved, such as the Hungate roll-tube technique, anaerobic chambers, anaerobic jars, and the use of antioxidant agents [55, 56]. This study increases the number of anaerobic species isolated at least once from humans [27] to 662 (23.85%) by adding 276 (45.7%) anaerobic species, representing around half of the currently added species (Additional file 1). Therefore, when looking at the distribution of the anaerobic bacteria across the entire repertoire (2776), there are a total of 459 species in the gut, of which 215 are reported in this study (Table 1).

## The revival of culture has paved the way for the exploration of the bacterial repertoire associated with human beings

### Approaches to discover new bacteria

Our current knowledge of the repertoire of human-associated bacteria remains incomplete and includes only 2776 cultured pathogenic and commensal bacteria [27]. Nevertheless, culture allowed us to isolate 94 bacterial species from clinical samples, of which 43 were novel and 51 were known species that had not been reported to be isolated from humans [57, 58]. Moreover, our laboratory succeeded in using culture to isolate 25 environmental species that have also recently been considered to be associated with humans. Of these, 23 were new [44]. We also reported the correlation of 18 *Rickettsia* sp. to human infections, following its detection in ticks and fleas. Additionally, the ability to culture fastidious microorganism led to a significant evolution in infectious diseases following the Koch postulate, such as the cases of *Tropheryma whipplei* [59–61] or *Bartonella* species [62–67], where its culture enhanced diagnosis as well as our knowledge in terms of its host interactions. Likewise, 11 *Mycobacterial* species were isolated, including five pathogenic strains (*M*. *barassiae*, *M*. *bolletii*, *M*. *conceptionense*, *M*. *massiliense*, and *M*. *tahitimassiliense*) [44]. The ability to isolate, rather than simply obtain sequences of bacterial species, will allow researchers to further analyze their biological significance, features, and therapeutic potentials.

Following the study by Hugon et al., in 2015 [27], our laboratory was able to isolate and report 288 new species from different human body sites using enriched culture-based methods. Two hundred thirty-two new species were isolated from the human gut, of which 163 were anaerobic and 69 belonged to genera that can tolerate oxygen. Likewise, of the 14 new species isolated from the urinary tract, three were aerobic and 11 anaerobic. As for vaginal microbiota, a total of 13 new species were isolated, of which 11 were anaerobic. Finally, 10 aerobic and three anaerobic new species were isolated from the respiratory system, two aerobic and one anaerobic species from the skin, two aerobic species from a human abscess, one anaerobic species and three aerobic species from the blood, two aerobic species from the cerebrospinal fluid, one aerobic species from a wound, one aerobic species from the peritoneal fluid, one aerobic species from a scalp pustule, two anaerobic species from the maternal colostrum, and one aerobic species from the foot of a patient with osteomyelitis.

The spectrum of these new species has now been added to the MALDI-TOF MS database at URMS database (http://www.mediterranee-infection.com/article.php?larub=280&titre=urms-database), being therefore available for clinical and microbiology laboratories using MS MALDI-TOF technology.

### Taxonogenomics to fasten the reporting of the new isolated species

As previously mentioned, culturomics has succeeded in reporting and isolating a significant number of new species. It is, therefore, mandatory to describe, at the different levels, the main characteristics of these species. To do so, a new taxonogenomic approach has recently been adopted by our laboratory [68]. Taxonogenomics targets the proteomic, phenotypic, and genotypic traits of the bacterial species. Firstly, a bacterial species is considered to be novel when its MALDI-TOF score is less than 2 and its 16S rRNA gene sequence similarity to the closest phylogenetic species is less than 98.7%. When this happens, its unique proteomic spectrum, generated by MALDI-TOF MS, is added to Bruker’s database and its 16S gene rRNA sequence is submitted in the NCBI nucleotide database for a faster identification in the future. The genome of the new bacterial species is then sequenced for annotation and comparison with close species in terms of DNA G+C content, size, gene content, percent of coding sequences, gene distribution in COG categories [69], number of genes coding for RNA, mobile genetic elements, transmembrane helices, signal peptides, and others if needed. The extent of genetic similarity between the compared bacterial isolates is also assessed by the Average of Genomic Identity of Orthologous Gene Sequences (AGIOS) and the establishment of the digital DDH with the help of the GGDC online software (http://ggdc.dsmz.de/distcalc2.php). New bacterial species are also tested for their ability to grow at different temperatures and environments, their resistance towards several common antibiotics, their sporulation ability, and their different biochemical characteristics.

**Fig. 5:**
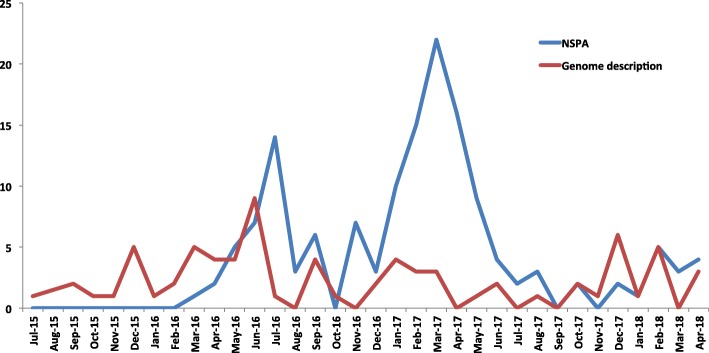
Number of reported species isolated for the first time in humans by culturomics using the new species announcement format compared to the full description format

This approach has enabled the description of over 146 new species, with an average of three species per month since April 2018. We now report new isolated species using a new format, known as the new species announcement (NSPA) [70]. The rate of identification shows no signs of plateau, and the NSPA has been proved to be efficient in terms of reporting when compared to the typical descriptive format. For instance, waiting for the complete description and validation of the name of the newly isolated species is considered to be far too long to be accessible to the scientific community when compared to NSPA, which takes around 3 months after submission to be published online. The NSPA reveals the isolation site of the new species, its growth conditions [70], the main phenotypic characteristics (gram, size, oxidase, and catalase), phylogenetic positioning among closely related species with standing in nomenclature, the MALDI-TOF spectrum, and the 16S rRNA gene sequences. Since the launch of the NSPA format in 2016, our laboratory has successfully reported over 100 novel species prior to their complete descriptions, with the average number of species reported per month being approximately three times higher than those in the standard descriptive papers. This enables faster communication of information within the scientific community, especially when, as previously mentioned, a significant number of novel species are reported by culturomics (Fig. 5).

## Prokaryotes in humans and their pathogenicity

Pathogenicity is a complex mechanism that depends on the ability of a microorganism to cause diseases due to several factors such as virulence, inoculum, and host factors. Defining a microorganism as “pathogenic” appears to be very difficult in several cases, since a commensal species might become pathogenic when the opportunity arises [71]. *Escherichia coli* is the perfect example; this bacterium is usually considered commensal when found in the human intestine but can become pathogenic if it reaches extra-colonic sites [72]. Similarly, polymicrobial and nosocomial infections are both agent- and host-dependent and should also be taken into account. *Staphylococcus aureus* is frequently detected during polymicrobial infections and can also play a commensal or even a protective role during infections [41, 42, 73]. In order to assess the pathogenicity of the reported bacteria in this repertoire, we adapted the same method as Hugon et al., grouping species based on their biological risk of infection [27]. Only 10 of the 604 species, added in this current study, were reported in the risk group database. Nine belonged to risk group 2, defined by a low community risk and a relative individual infection risk, and one belonged to risk group 3, defined by a low community risk and a high individual risk.

The lack of information towards the pathogenicity of the remaining species could be explained by the fact that most of them were new species that had not previously been isolated or had been isolated for the first time recently in humans. This fact remains of concern and highlights the need to create a database of all pathogenic bacteria and clinical cases that may be reported in the literature but are not yet easily accessible. Finally, in order to get an idea about the clinical occurrence of the species reported in our repertoire, which might represent some level of awareness in terms of virulence and pathogenicity, we investigated the PubMed database for articles reporting its isolation in clinical samples or clinical cases using an advanced search for articles containing the name of the bacteria (with its synonyms) with clinical, patient, or disease. Excluding new species isolated by culturomics, 148 species were reported to have been isolated in clinical specimens or clinical cases, of which eight were the cause of bacteremia (Additional files 1 and 2). After examining the isolated clinical species, we found that 58 (39%) were novel and had not been previously reported nor isolated from humans or from any other site. In addition, in 2017, Lagier et al. reported the isolation and identification of 12 bacterial species through culturomics, under different pathogenic conditions [44]. This prompted us to continue describing the human microbiota using culturomics, thus demonstrating its effective contribution towards clinical sample evaluations [26]. The fact that such a significant number of new species were detected in a clinical context highlights the need to move beyond the notion that “one bacteria causes one disease” [74]. As a perspective, high-throughput sequencing and extensive culture methods applied on clinical specimen could substantially expand the spectrum of prokaryotes involved in infectious diseases.

## Metagenomics and culturomics: complementarity rather than contradiction

Human microbiota communities have been largely studied by metagenomics, which succeeded in correlating its content and diversity to health and diseases. For example, acne has been linked to alterations in the cutaneous microbiome [75] in addition to the dominance of *Firmicutes* relative to *Actinobacteria*, in psoriatic lesions [6]. Furthermore, the detection of *Helicobacter pylori* in the gastric microbiota has been correlated with age-related diseases [7] and has been found to increase the risk of developing several health issues, including gastric adenocarcinoma and peptic ulcers [8]. Colonic microbiota are currently being studied to correlate changes with colorectal cancer [9], and the gut microbiome has been suggested to play an important role in inflammatory bowel diseases such as Crohn’s disease, where a decrease in obligate anaerobes belonging to the *Firmicutes* phylum was observed, along with an increase in facultative anaerobes belonging to the *Enterobacteriaceae* family [10]. Moreover, gut microbiota composition may be involved in hepatologic conditions such as non-alcoholic fatty liver disease (NAFLD) [11], besides having a certain role in the obesity phenotype [12, 13]. All these examples have led to the mass analyses of the bacterial content of the microbiota during diseases and have helped to create hypotheses for researchers on the possibility of developing new bacteriotherapy approaches, which can only be achieved whenever the different species taken into consideration are cultured and available for further experimentations [74].

Indeed, intensive culture methods have led the discovery of human commensals, which were thereafter found to be microbes of medical importance in high-throughput sequencing-based analyses, making both methods complementary. For instance, the depletion of *Faecalibacterium prausnitzii* was correlated with inflammatory bowel diseases (IBD), including colitis and Crohn’s disease [76]. In addition, *Akkermansia muciniphila*, cultivated only in 2004 from the human gut [77], is now considered a probiotic [78] as many metagenomic studies have demonstrated its association with metabolic disorders such as obesity or diabetes [14, 79]. Moreover, using a simplified culture workflow, the role of *Clostridium butyricum* in necrotizing enterocolitis was demonstrated by a case study using culturomics [80]. This points out that culture and metagenomic approaches should be grasped in a circular pattern, even though culture-based microbiota studies are difficult to apply to an entire cohort and time consuming.

This complementarity has also been demonstrated in an elegant study dedicated to show the influence of the gut microbiota composition on the efficiency of PD-1-based immunotherapy against several tumors [81]. While shotgun sequencing of fecal microbiota evidenced overrepresentation of *Akkermansia muciniphila* in responders when compared to non-responders, the culturomics approach highlighted an association between the presence of *Enterococcus hirae* and the response to PD-1 treatment. Of note, a previous strain of *Enterococcus hirae*, currently considered an oncobiotic, was cultivated from mouse spleen nodes [16]. Finally, the proof of concept of this work was definitely established in a murine model, in which an association of *E*. *hirae and A*. *muciniphila* bacterial strains were administered. As a result, efficacy of PD-1 treatment was reordered in mice previously receiving antibiotics.

## Challenging operational taxonomic units

As previously mentioned, when it comes to the molecular profiling of the human microbiome, OTUs are one of the major challenges. OTUs are simply the results of the higher diversity obtained through the description of the human microbiome by metagenomics [33, 34]. However, the higher microbial diversity in molecular methods which results in a significant number of OTUs does not pose any difficulties, particularly as its reference bacteria can be cultured when the proper conditions are met [18]. In fact, significant efforts have been recently made to reveal the identity of the OTUs and to decipher the dark matter of the human microbiota using culturomics. This has succeeded in demonstrating the ability of culture to report a significant proportion of the human gut microbiota, while maintaining its overall community structure [26, 82–84]. Similarly, in a study using the concept of the culture-enriched molecular profiling of the human gut, the ability of culture to recover the majority of OTUs when after-culture sequencing was implemented has been shown, thus confirming that culture is not only complementary to molecular approaches but is also essential to the description of the human microbiome [85].

## Conclusion

The study of the human microbiota is more than ever a major challenge as its implication in health and diseases has exceeded our expectations. For instance, the concept that human microbes can modulate response to anticancer therapy [16, 81] or be involved in HIV transmission [86] was difficult to imagine few years ago. These substantial advances were made possible thanks to the conjoint advances in culture, sequencing methods, and bioinformatic analysis. Herein, we highlight the considerable efforts recently made for culturing new microbes. Thus, a total of 2776 species isolated from the human body at different sites has been reached, of which 604 are reported in the present work. This represents a substantial increase of 28% within only 3 years. Culturomics contributed up to 66.2% in updating the previous repertoire and demonstrated the fundamental role of commensal culturing in describing and unveiling the hidden part of the human microbiota [26]. Beyond the impact of culturomics, this substantial increase is part of the current rebirth of culture in the field of microbiology that we are witnessing. As a matter of fact, Browne et al. recently cultured 68 new taxa, including new families and genera, while cultivating fecal specimen from six healthy individuals [87]. To the best of our knowledge, these taxa were not yet characterized. Interestingly, 37 of these new taxa belong, with different degrees of priority, to the “most wanted taxa” list. The latter was established as a part of the Human Microbiome Project (HMP) following characterization of bacterial communities at several body sites among 200 healthy volunteers and corresponded to the organisms of which its genome sequences are missing [88]. These microbes are underrepresented in culture collections, and we believe that high-throughput culture methods should, with time, enable their recovery. This points out the need of an updated repertoire of the bacteria cultured from the human beings but also raises several issues. Indeed, the pace of the human new species isolation in microbiota descriptive studies has recently accelerated, as shown in this study, but the delay of its genome’s integration into the available genomic databases is incompatible with the intense research dedicated in this field. Even if we have accelerated the publication of these new isolated taxa through the new species announcement format, the time for official validation of these new species slows its integration despite having custom databases that can be built to capture the optimal diversity among assigned sequences [26]. This also evidences the need to include genome sequencing as a part of the description of new species, at least if they were human isolated.

Taken together, these elements should encourage keeping a repertoire of prokaryotes associated with human beings real-time updated. If the vast majority of the bacteria newly isolated are in fact commensals, some can be further recovered as pathogens, as exemplified by *Akkermansia muciniphila* which has been recently isolated from blood cultures. Only time will allow adjudicating whether prokaryotes included in such a repertoire should be considered as commensals, pathogens, or bacteria passing through the human being and thus allowing a better interpretation of human microbiome studies [89].

## Additional files

Additional file 1: General characteristics of the reported species in this repertoire along with its classification based on oxygen tolerance (0 being able to tolerate oxygen and 1 strictly anaerobic), risk group, potential pathogenicity, and the PMID of the article. A: New species isolated by culturomics approach but not in the gut. B: Species isolated without culturomics approach and not isolated in the gut. C: New species isolated not using culturomics approach. D: Species isolated by routine culture in our laboratory. E: Species isolated in the human gut by culturomics. F: New species isolated in the human gut by culturomics. G: Species isolated in the human gut and without using culturomics. (XLSX 40 kb)

Additional file 2: This table represents, in sheet 1, the taxonomic classification of the bacterial species according to NCBI taxonomy (https://www.ncbi.nlm.nih.gov/Taxonomy/taxonomyhome.html/) and, in sheet 2, all the species classification according to their phylum and that have not been added yet in NCBI taxonomy database. (XLSX 514 kb)

Additional file 3: This table represents all the bacterial species isolated at least once from the human being, classified according to its phylum and combining the present work and the previous species reported by Hugon et al., in 2015. (XLSX 73 kb)

## References

[1] Whitman WB, Coleman DC, Wiebe WJ (1998). Prokaryotes: the unseen majority. Proc Natl Acad Sci U S A.

[2] Antranikian G, Vorgias CE, Bertoldo C (2005). Extreme environments as a resource for microorganisms and novel biocatalysts. Adv Biochem Eng Biotechnol.

[3] Horner-Devine MC, Carney KM, Bohannan BJM (2004). An ecological perspective on bacterial biodiversity. Proc Biol Sci.

[4] Curtis TP, Sloan WT, Scannell JW (2002). Estimating prokaryotic diversity and its limits. Proc Natl Acad Sci U S A.

[5] Locey KJ, Lennon JT (2016). Scaling laws predict global microbial diversity. Proc Natl Acad Sci U S A.

[6] Patel RV, Lebwohl M (2011). In the clinic. Psoriasis. Ann Intern Med.

[7] Gao Z, Tseng C, Strober BE, Pei Z, Blaser MJ (2008). Substantial alterations of the cutaneous bacterial biota in psoriatic lesions. PLoS One.

[8] Atherton JC, Blaser MJ (2009). Coadaptation of *Helicobacter pylori* and humans: ancient history, modern implications. J Clin Invest.

[9] McColl KEL (2010). Clinical practice. *Helicobacter pylori* infection. N Engl J Med.

[10] Plottel CS, Blaser MJ (2011). Microbiome and malignancy. Cell Host Microbe.

[11] Rigottier-Gois L (2013). Dysbiosis in inflammatory bowel diseases: the oxygen hypothesis. ISME J.

[12] Abu-Shanab A, Quigley EMM (2010). The role of the gut microbiota in nonalcoholic fatty liver disease. Nat Rev Gastroenterol Hepatol.

[13] Turnbaugh PJ, Ley RE, Mahowald MA, Magrini V, Mardis ER, Gordon JI (2006). An obesity-associated gut microbiome with increased capacity for energy harvest. Nature.

[14] Kobyliak N, Conte C, Cammarota G, Haley AP, Styriak I, Gaspar L, et al. Probiotics in prevention and treatment of obesity: a critical view. Nutr Metab [Internet]. 2016;13 Available from: http://www.nutritionandmetabolism.com/content/13/1/14. Cited 11 Jun 2017 10.1186/s12986-016-0067-0 PMC4761174 26900391

[15] Vétizou M, Pitt JM, Daillère R, Lepage P, Waldschmitt N, Flament C (2015). Anticancer immunotherapy by CTLA-4 blockade relies on the gut microbiota. Science.

[16] Daillère R, Vétizou M, Waldschmitt N, Yamazaki T, Isnard C, Poirier-Colame V (2016). *Enterococcus hirae* and *Barnesiella intestinihominis* facilitate cyclophosphamide-induced therapeutic immunomodulatory effects. Immunity.

[17] Million M, Tidjani Alou M, Khelaifia S, Bachar D, Lagier J-C, Dione N, et al. Increased gut redox and depletion of anaerobic and methanogenic prokaryotes in severe acute malnutrition. Sci Rep [Internet]. 2016;6 Available from: http://www.nature.com/articles/srep26051. Cited 11 Jun 2017 10.1038/srep26051 PMC4869025 27183876

[18] Lagier J-C, Hugon P, Khelaifia S, Fournier P-E, La Scola B, Raoult D (2015). The rebirth of culture in microbiology through the example of culturomics to study human gut microbiota. Clin Microbiol Rev.

[19] Lagier J-C, Million M, Hugon P, Armougom F, Raoult D (2012). Human gut microbiota: repertoire and variations. Front Cell Infect Microbiol.

[20] Loman NJ, Constantinidou C, Christner M, Rohde H, Chan JZ-M, Quick J (2013). A culture-independent sequence-based metagenomics approach to the investigation of an outbreak of Shiga-toxigenic *Escherichia coli* O104:H4. JAMA.

[21] Singh P, Teal TK, Marsh TL, Tiedje JM, Mosci R, Jernigan K (2015). Intestinal microbial communities associated with acute enteric infections and disease recovery. Microbiome.

[22] Greub G (2012). Culturomics: a new approach to study the human microbiome. Clin Microbiol Infect Off Publ Eur Soc Clin Microbiol Infect Dis.

[23] Dubourg G, Lagier JC, Armougom F, Robert C, Hamad I, Brouqui P (2013). The gut microbiota of a patient with resistant tuberculosis is more comprehensively studied by culturomics than by metagenomics. Eur J Clin Microbiol Infect Dis.

[24] Pfleiderer A, Lagier J-C, Armougom F, Robert C, Vialettes B, Raoult D (2013). Culturomics identified 11 new bacterial species from a single anorexia nervosa stool sample. Eur J Clin Microbiol Infect Dis.

[25] Dubourg G, Lagier JC, Robert C, Armougom F, Hugon P, Metidji S (2014). Culturomics and pyrosequencing evidence of the reduction in gut microbiota diversity in patients with broad-spectrum antibiotics. Int J Antimicrob Agents.

[26] Lagier J-C, Khelaifia S, Alou MT, Ndongo S, Dione N, Hugon P (2016). Culture of previously uncultured members of the human gut microbiota by culturomics. Nat Microbiol.

[27] Hugon P, Dufour J-C, Colson P, Fournier P-E, Sallah K, Raoult D (2015). A comprehensive repertoire of prokaryotic species identified in human beings. Lancet Infect Dis.

[28] Ley RE, Turnbaugh PJ, Klein S, Gordon JI (2006). Microbial ecology: human gut microbes associated with obesity. Nature.

[29] Ley RE, Bäckhed F, Turnbaugh P, Lozupone CA, Knight RD, Gordon JI (2005). Obesity alters gut microbial ecology. Proc Natl Acad Sci U S A.

[30] Gill SR, Pop M, Deboy RT, Eckburg PB, Turnbaugh PJ, Samuel BS (2006). Metagenomic analysis of the human distal gut microbiome. Science.

[31] Turnbaugh PJ, Ley RE, Hamady M, Fraser-Liggett CM, Knight R, Gordon JI (2007). The human microbiome project. Nature.

[32] Gossling J, Slack JM (1974). Predominant gram-positive bacteria in human feces: numbers, variety, and persistence. Infect Immun.

[33] Eckburg PB, Bik EM, Bernstein CN, Purdom E, Dethlefsen L, Sargent M (2005). Diversity of the human intestinal microbial flora. Science.

[34] Hayashi H, Sakamoto M, Benno Y (2002). Phylogenetic analysis of the human gut microbiota using 16S rDNA clone libraries and strictly anaerobic culture-based methods. Microbiol Immunol.

[35] Wilson KH, Blitchington RB (1996). Human colonic biota studied by ribosomal DNA sequence analysis. Appl Environ Microbiol.

[36] Lagier J-C, Armougom F, Million M, Hugon P, Pagnier I, Robert C (2012). Microbial culturomics: paradigm shift in the human gut microbiome study. Clin Microbiol Infect Off Publ Eur Soc Clin Microbiol Infect Dis.

[37] Cook GM, Berney M, Gebhard S, Heinemann M, Cox RA, Danilchanka O, et al. Physiology of *Mycobacteria*. Adv Microb Physiol [Internet]. 2009:81–319. Elsevier. Available from: http://linkinghub.elsevier.com/retrieve/pii/S0065291109055027. Cited 11 Jun 2017 10.1016/S0065-2911(09)05502-7 PMC3728839 19573696

[38] Pinheiro RO, de Souza Salles J, Sarno EN, Sampaio EP (2011). *Mycobacterium leprae*? Host-cell interactions and genetic determinants in leprosy: an overview. Future Microbiol.

[39] Whipps CM, Tortoli E, Matsumoto CK, Martin A, Droz S, Leão SC (2015). *Mycobacterium saopaulense* sp. nov., a rapidly growing mycobacterium closely related to members of the *Mycobacterium chelonae*? *Mycobacterium abscessus* group. Int J Syst Evol Microbiol.

[40] Adékambi T, Berger P, Raoult D, Drancourt M (2006). rpoB gene sequence-based characterization of emerging non-tuberculous mycobacteria with descriptions of *Mycobacterium bolletii* sp. nov., *Mycobacterium phocaicum* sp. nov. and *Mycobacterium aubagnense* sp. nov. Int J Syst Evol Microbiol.

[41] Ben Salah I, Cayrou C, Raoult D, Drancourt M (2009). *Mycobacterium marseillense* sp. nov., *Mycobacterium timonense* sp. nov. and *Mycobacterium bouchedurhonense* sp. nov., members of the *Mycobacterium avium* complex. Int J Syst Evol Microbiol.

[42] Adékambi T, Stein A, Carvajal J, Raoult D, Drancourt M (2006). Description of *Mycobacterium conceptionense* sp. nov., a *Mycobacterium fortuitum* group organism isolated from a posttraumatic osteitis inflammation. J Clin Microbiol.

[43] Adékambi T, Reynaud-Gaubert M, Greub G, Gevaudan M-J, La Scola B, Raoult D (2004). Amoebal coculture of “*Mycobacterium massiliense*” sp. nov. from the sputum of a patient with hemoptoic pneumonia. J Clin Microbiol.

[44] Lagier JC, Drancourt M, Charrel R, Bittar F, Ranque S, Raoult D. Many more microbes in humans: enlarging the microbiome repertoire. Clin Infect Dis Press. 2017;65:S20–9. 10.1093/cid/cix404 28859350

[45] Nouioui I, Carro L, Teramoto K, Igual JM, Jando M, Del Carmen Montero-Calasanz M (2017). *Mycobacterium eburneum* sp. nov., a non-chromogenic, fast-growing strain isolated from sputum. Int J Syst Evol Microbiol.

[46] Paniz-Mondolfi AE, Greninger AL, Ladutko L, Brown-Elliott BA, Vasireddy R, Jakubiec W (2017). *Mycobacterium grossiae* sp. nov., a rapidly growing, scotochromogenic species isolated from human clinical respiratory and blood culture specimens. Int J Syst Evol Microbiol.

[47] Shahraki AH, Trovato A, Mirsaeidi M, Borroni E, Heidarieh P, Hashemzadeh M (2017). *Mycobacterium persicum* sp. nov., a novel species closely related to *Mycobacterium kansasii* and *Mycobacterium gastri*. Int J Syst Evol Microbiol.

[48] Davidson RM, DeGroote MA, Marola JL, Buss S, Jones V, McNeil MR (2017). *Mycobacterium talmoniae* sp. nov., a slowly growing mycobacterium isolated from human respiratory samples. Int J Syst Evol Microbiol.

[49] Hugon P, Lagier J-C, Robert C, Lepolard C, Papazian L, Musso D (2013). Molecular studies neglect apparently gram-negative populations in the human gut microbiota. J Clin Microbiol.

[50] Li J, Jia H, Cai X, Zhong H, Feng Q, Sunagawa S (2014). An integrated catalog of reference genes in the human gut microbiome. Nat Biotechnol.

[51] Human Microbiome Project Consortium (2012). A framework for human microbiome research. Nature.

[52] Human Microbiome Project Consortium (2012). Structure, function and diversity of the healthy human microbiome. Nature.

[53] Stokes EJ (1975). Anaerobic bacteria: role in disease. J Clin Pathol.

[54] DiBaise JK, Zhang H, Crowell MD, Krajmalnik-Brown R, Decker GA, Rittmann BE (2008). Gut microbiota and its possible relationship with obesity. Mayo Clin Proc.

[55] La Scola B, Khelaifia S, Lagier J-C, Raoult D (2014). Aerobic culture of anaerobic bacteria using antioxidants: a preliminary report. Eur J Clin Microbiol Infect Dis Off Publ Eur Soc Clin Microbiol.

[56] Summanen PH, McTeague M, Väisänen ML, Strong CA, Finegold SM (1999). Comparison of recovery of anaerobic bacteria using the Anoxomat, anaerobic chamber, and GasPak jar systems. Anaerobe.

[57] Seng P, Abat C, Rolain JM, Colson P, Lagier J-C, Gouriet F (2013). Identification of rare pathogenic bacteria in a clinical microbiology laboratory: impact of matrix-assisted laser desorption ionization-time of flight mass spectrometry. J Clin Microbiol.

[58] Drancourt M, Berger P, Raoult D (2004). Systematic 16S rRNA gene sequencing of atypical clinical isolates identified 27 new bacterial species associated with humans. J Clin Microbiol.

[59] Fenollar F, Lagier J-C, Raoult D (2014). *Tropheryma whipplei* and Whipple’s disease. J Inf Secur.

[60] Raoult D, Birg ML, Scola BL, Fournier PE, Enea M, Lepidi H (2000). Cultivation of the Bacillus of Whipple’s disease. N Engl J Med.

[61] Rolain J-M, Fenollar F, Raoult D (2007). False positive PCR detection of *Tropheryma whipplei* in the saliva of healthy people. BMC Microbiol.

[62] Okaro U, Addisu A, Casanas B, Anderson B (2017). Bartonella species, an emerging cause of blood-culture-negative endocarditis. Clin Microbiol Rev.

[63] Slater LN, Welch DF, Hensel D, Coody DW (1990). A newly recognized fastidious gram-negative pathogen as a cause of fever and bacteremia. N Engl J Med.

[64] Perkocha LA, Geaghan SM, Yen TS, Nishimura SL, Chan SP, Garcia-Kennedy R (1990). Clinical and pathological features of bacillary peliosis hepatis in association with human immunodeficiency virus infection. N Engl J Med.

[65] Relman DA, Loutit JS, Schmidt TM, Falkow S, Tompkins LS (1990). The agent of bacillary angiomatosis. An approach to the identification of uncultured pathogens. N Engl J Med.

[66] Welch DF, Pickett DA, Slater LN, Steigerwalt AG, Brenner DJ (1992). *Rochalimaea henselae* sp. nov., a cause of septicemia, bacillary angiomatosis, and parenchymal bacillary peliosis. J Clin Microbiol.

[67] Brenner DJ, O’Connor SP, Winkler HH, Steigerwalt AG (1993). Proposals to unify the genera *Bartonella* and *Rochalimaea*, with descriptions of *Bartonella quintana* comb. nov., *Bartonella vinsonii* comb. nov., *Bartonella henselae* comb. nov., and *Bartonella elizabethae* comb. nov., and to remove the family *Bartonellaceae* from the order Rickettsiales. Int J Syst Bacteriol.

[68] Ramasamy D, Mishra AK, Lagier J-C, Padhmanabhan R, Rossi M, Sentausa E (2014). A polyphasic strategy incorporating genomic data for the taxonomic description of novel bacterial species. Int J Syst Evol Microbiol.

[69] Abdallah RA, Beye M, Diop A, Bakour S, Raoult D, Fournier P-E. The impact of culturomics on taxonomy in clinical microbiology. Antonie Van Leeuwenhoek [Internet]. 2017; Available from: http://link.springer.com/10.1007/s10482-017-0871-1. Cited 11 Jun 2017 10.1007/s10482-017-0871-1 28389704

[70] Fournier P-E, Raoult D, Drancourt M (2016). Republication of «new species announcement», a new format to prompt the description of new human microbial species. Hum Microbiome J.

[71] Isenberg HD (1988). Pathogenicity and virulence: another view. Clin Microbiol Rev.

[72] Kaper JB, Nataro JP, Mobley HL (2004). Pathogenic *Escherichia coli*. Nat Rev Microbiol.

[73] Sotto A, Richard J-L, Messad N, Molinari N, Jourdan N, Schuldiner S (2012). Distinguishing colonization from infection with *Staphylococcus aureus* in diabetic foot ulcers with miniaturized oligonucleotide arrays. Diabetes Care.

[74] Lagier J-C, Dubourg G, Amrane S, Raoult D (2017). Koch postulate: why should we grow bacteria?. Arch Med Res.

[75] Fournier P-E, Drancourt M (2015). New microbes new infections promotes modern prokaryotic taxonomy: a new section “TaxonoGenomics: new genomes of microorganisms in humans”. New Microbes New Infect.

[76] Sokol H, Pigneur B, Watterlot L, Lakhdari O, Bermúdez-Humarán LG, Gratadoux J-J (2008). *Faecalibacterium prausnitzii* is an anti-inflammatory commensal bacterium identified by gut microbiota analysis of Crohn disease patients. Proc Natl Acad Sci U S A.

[77] Derrien M, Vaughan EE, Plugge CM, de Vos WM (2004). *Akkermansia muciniphila* gen. nov., sp. nov., a human intestinal mucin-degrading bacterium. Int J Syst Evol Microbiol.

[78] Gómez-Gallego C, Pohl S, Salminen S, De Vos WM, Kneifel W (2016). *Akkermansia muciniphila*: a novel functional microbe with probiotic properties. Benef Microbes.

[79] Dao MC, Everard A, Aron-Wisnewsky J, Sokolovska N, Prifti E, Verger EO (2016). *Akkermansia muciniphila* and improved metabolic health during a dietary intervention in obesity: relationship with gut microbiome richness and ecology. Gut.

[80] Cassir N, Benamar S, Khalil JB, Croce O, Saint-Faust M, Jacquot A (2015). *Clostridium butyricum* strains and dysbiosis linked to necrotizing enterocolitis in preterm neonates. Clin Infect Dis Off Publ Infect Dis Soc Am.

[81] Routy B, Le Chatelier E, Derosa L, Duong CPM, Alou MT, Daillère R (2018). Gut microbiome influences efficacy of PD-1-based immunotherapy against epithelial tumors. Science.

[82] Sommer MOA (2015). Advancing gut microbiome research using cultivation. Curr Opin Microbiol.

[83] Goodman AL, Kallstrom G, Faith JJ, Reyes A, Moore A, Dantas G (2011). Extensive personal human gut microbiota culture collections characterized and manipulated in gnotobiotic mice. Proc Natl Acad Sci U S A.

[84] Rettedal EA, Gumpert H, Sommer MOA (2014). Cultivation-based multiplex phenotyping of human gut microbiota allows targeted recovery of previously uncultured bacteria. Nat Commun.

[85] Lau JT, Whelan FJ, Herath I, Lee CH, Collins SM, Bercik P, et al. Capturing the diversity of the human gut microbiota through culture-enriched molecular profiling. Genome Med [Internet]. 2016;8 Available from: http://genomemedicine.biomedcentral.com/articles/10.1186/s13073-016-0327-7. Cited 11 Jun 2017 10.1186/s13073-016-0327-7 PMC4929786 27363992

[86] McClelland RS, Lingappa JR, Srinivasan S, Kinuthia J, John-Stewart GC, Jaoko W, et al. Evaluation of the association between the concentrations of key vaginal bacteria and the increased risk of HIV acquisition in African women from five cohorts: a nested case-control study. Lancet Infect Dis. 2018;18:554–64 10.1016/S1473-3099(18)30058-6 PMC6445552 29396006

[87] Browne HP, Forster SC, Anonye BO, Kumar N, Neville BA, Stares MD (2016). Culturing of “unculturable” human microbiota reveals novel taxa and extensive sporulation. Nature.

[88] Fodor AA, DeSantis TZ, Wylie KM, Badger JH, Ye Y, Hepburn T, et al. The “most wanted” taxa from the human microbiome for whole genome sequencing. PLoS ONE [Internet]. 2012;7 Available from: https://www.ncbi.nlm.nih.gov/pmc/articles/PMC3406062/10.1371/journal.pone.0041294 PMC3406062 22848458

[89] Lagier JC, Dubourg G, Million M, Cadoret F, Bilen M, Fenollar F, et al. Culturing human microbiota and culturomics. Nat Rev Microbiol. 4 In Press 10.1038/s41579-018-0041-0 29937540

